# Targeting bladder function with network-specific epidural stimulation after chronic spinal cord injury

**DOI:** 10.1038/s41598-022-15315-2

**Published:** 2022-07-01

**Authors:** April N. Herrity, Sevda C. Aslan, Samineh Mesbah, Ricardo Siu, Karthik Kalvakuri, Beatrice Ugiliweneza, Ahmad Mohamed, Charles H. Hubscher, Susan J. Harkema

**Affiliations:** 1 Kentucky Spinal Cord Injury Research Center, Department of Neurological Surgery, The University of Louisville, 220 Abraham Flexner Way, Suite 1518, Louisville, KY 40202 USA; 2 Department of Neurological Surgery, University of Louisville, Louisville, KY USA; 3 Department of Physiology, University of Louisville, Louisville, KY USA; 4 Department of Health Sciences, University of Louisville, Louisville, KY USA; 5 Department of Urology, University of Louisville, Louisville, KY USA; 6 Department of Anatomical Sciences and Neurobiology, University of Louisville, Louisville, KY USA

**Keywords:** Neuroscience, Diseases of the nervous system

## Abstract

Profound dysfunctional reorganization of spinal networks and extensive loss of functional continuity after spinal cord injury (SCI) has not precluded individuals from achieving coordinated voluntary activity and gaining multi-systemic autonomic control. Bladder function is enhanced by approaches, such as spinal cord epidural stimulation (scES) that modulates and strengthens spared circuitry, even in cases of clinically complete SCI. It is unknown whether scES parameters specifically configured for modulating the activity of the lower urinary tract (LUT) could improve both bladder storage and emptying. Functional bladder mapping studies, conducted during filling cystometry, identified specific scES parameters that improved bladder compliance, while maintaining stable blood pressure, and enabled the initiation of voiding in seven individuals with motor complete SCI. Using high-resolution magnetic resonance imaging and finite element modeling, specific neuroanatomical structures responsible for modulating bladder function were identified and plotted as heat maps. Data from this pilot clinical trial indicate that scES neuromodulation that targets bladder compliance reduces incidences of urinary incontinence and provides a means for mitigating autonomic dysreflexia associated with bladder distention. The ability to initiate voiding with targeted scES is a key step towards regaining volitional control of LUT function, advancing the application and adaptability of scES for autonomic function.

## Introduction

Neurogenic bladder dysfunction is highly prevalent following a spinal cord injury (SCI)^1^, profoundly impacting health and quality of life^2,3^. Loss of volitional control of micturition, consistent with an upper motor neuron-type injury, is accompanied by detrusor overactivity and detrusor-sphincter dyssynergia, where simultaneous detrusor and urinary sphincter contractions lead to high bladder pressure and insufficient emptying^4^. Major urological concerns contributing to increased morbidity and mortality include incontinence, repeated lower urinary tract (LUT) infections that can result in sepsis, chronic vesicoureteral reflux, and hydronephrosis with progression to renal insufficiency^5,6^. Furthermore, SCI above the sixth thoracic vertebra (T6) impairs cardiovascular reflexes, leading to autonomic dysreflexia (sudden elevation of blood pressure greater than 20 mmHg above one’s usual baseline^7^) that limits bladder storage^8^. Standard management of LUT dysfunction post-SCI includes a combination of pharmacological and catheterization approaches for storage and emptying, respectively, or insertion of an indwelling catheter when hand function is limited. While these measures preserve upper tract function, they do not address the potential to regain LUT control and further independence over time.

Restoration of bladder function is rated as a high priority among individuals with SCI^9,10^. A recent survey investigating consumer needs and priorities indicates a strong desire and willingness to adopt neuromodulation interventions to facilitate a return to more normal bladder function and help reduce secondary complications negatively impacting quality of life^11^. Lumbosacral spinal cord epidural stimulation (scES) combined with intensive activity-based recovery training is one such neuromodulatory approach that re-engages existing spinal circuits below the level of injury, promoting the novel post-injury circuitry to reorganize in functionally and physiologically significant ways^12–17^. Capitalizing on the inherent functional capacity that comprises these systemic circuits, scES enables autonomic circuits to recover significant levels of function^18–27^. We have previously shown that scES can be used to augment the lumbosacral neural circuitry below the level of injury sufficient to potentiate gains in bladder function^21^ achieved through activity-based recovery interventions alone^8,28^. Driven in part by consumer demand as well as from a paradigm shift in rehabilitative strategies focusing on a return to pre-injury function, there is a critical need for a therapeutic intervention that aims to restore normal or even partial LUT function. The standard of care, which includes anticholinergic therapy and chronic catheterization, has high rates of discontinuation^29^ and an increased chance of diminishing bladder compliance with time (indwelling catheters)^30^, respectively. Both approaches require life-long maintenance and have adverse side effects leading to recurring illness and reduced quality of life^31^. Importantly, cardiovascular complications associated with autonomic dysregulation post-SCI directly interfere with the ability to recover bladder function. Further development of the parameters of stimulation and programming strategies and protocols for improving bladder control and managing interactions from the cardiovascular system are also needed to advance neuromodulatory approaches.

Similar to scES, other non-surgical neuromodulation approaches, such as transcutaneous spinal cord stimulation can target multiple systems (motor and autonomic) by the select placement of the electrodes along the length of the spinal cord^32–39^. Pilot studies with transcutaneous stimulation applied over interspinous segments of T11 and L1 during urodynamics indicate improvements in bladder storage and emptying in individuals with motor/sensory complete through motor incomplete SCI^32,39^. Transcutaneous magnetic stimulation is another non-invasive approach, which when applied over the thoracolumbar spine for 16 weeks, enabled volitional voiding with significantly lower post-void residual volumes and a decreased need for daily self-catheterization in 5 subjects with motor complete SCI^40^. Transcutaneous stimulation in general may also be effective in identifying those individuals who respond to spinal cord stimulation, should device implantation be a future option as well as testing and determining the optimal spinal locations for different autonomic functions. From a clinical perspective, identifying those individuals who will potentially benefit from stimulation as well as establishing clear guidelines and protocols regarding the optimization and long-term adjustments of stimulation paradigms have been identified as critical areas to advancing the technology^41^. In the current pilot trial, LUT function and blood pressure responses to bladder distention were examined throughout a targeted scES mapping study on an initial cohort of individuals (n = 7). Appropriately selected stimulation parameters identified through scES bladder mapping were found to modulate local spinal reflexes important for both the maintenance of urinary continence and the initiation of voiding. Mapping of the overlapping 5–6–5 paddle array on each participant’s reconstructed 3-D spinal cord was also performed to better understand the inherent anatomical variability of the vertebral column with respect to the lumbosacral enlargement and location of the conus tip across participants. Finite element modeling was conducted to quantify the current density and distribution patterns generated by specific bladder cohorts, identifying the spinal cord locations optimal for modulating bladder storage and emptying. The selectivity and depth for targeting neural structures by scES as well as identifying the anatomical variability between individuals are critical to personalizing neuromodulatory strategies for paralysis. Thus, we hypothesized that targeted scES bladder mapping was necessary to access participant specific spinal networks in order to promote individual gains in bladder continence and micturition reflexes.

## Results

### Clinical characteristics

The clinical and demographic information for enrolled research participants is provided in Table 1. Characteristics represented in the table were determined from the time at which each participant enrolled in the study. Participant ages ranged from 26 to 39 years of age (32.1 ± 4.6), with a 6:1 ratio of males to females and an average time since injury at 9.1 ± 2.5 years. All participants were assessed as motor complete SCI, with the level of injury ranging from C3–T2. Three participants managed their bladders with a suprapubic (SP) catheter and four participants performed clean intermittent catheterization (CIC).

**Table 1:** Participant characteristics.

Participant ID	Age (years)	Sex	Neuro level	AIS grade	Time post injury (years)	Anal sensation	Bladder emptying method
A101	32	M	C3	A	9	No	SP
A96	27	F	C4	A	5	No	SP
B23	36	M	C5	B	8	Yes	SP
B21	33	M	C4	B	11	Yes	CIC
B24	26	M	C7	B	8	Yes	CIC
A68	39	M	C8	B	10	Yes	CIC
B07	32	M	T2	B	13	Yes	CIC

*AIS* American Spinal Injury Association Impairment Scale, *CIC* clean intermittent catheterization, *SP* suprapubic catheter.

### Bladder mapping—storage phase

**Figure 1:**
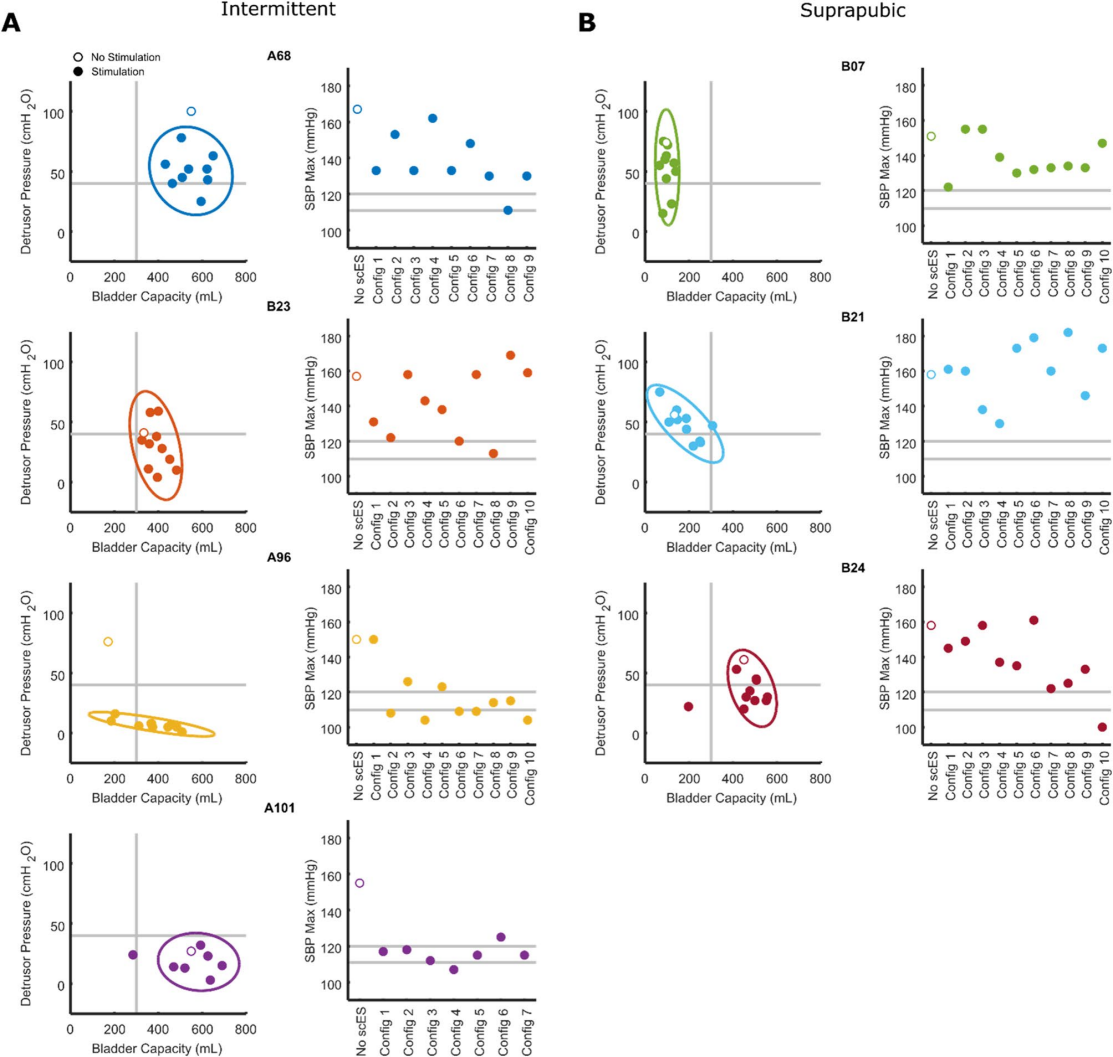
Scatter plots of the pressure–volume measurements obtained during urodynamics without scES (open circles) and from mapping with BC-scES (filled in circles) in participants using intermittent catheterization (**A**), n = 4, and in those using suprapubic catheters (**B**), n = 3. Ellipses show the 95% confidence interval for each participant after removing outliers. Vertical and horizontal lines indicate normative thresholds for minimum bladder capacity and maximum detrusor pressure, respectively. Blood pressure responses at maximum capacity for each participant are displayed next to each corresponding pressure–volume plot with optimal ranges targeted between 110–120 mmHg denoted by double horizontal lines. Note the shift toward normative bladder capacity, bladder pressure, and/or blood pressure with scES. *BC-scES* bladder compliance spinal cord epidural stimulation, *cmH*_*2*_*O* centimeters of water, *mL* milliliters, *mmHg* millimeters of mercury.

Maximum cystometric capacity values and corresponding detrusor and systolic blood pressure values attained without scES (open circles) and during bladder compliance scES mapping (BC-scES) sessions (closed circles) are plotted for those performing CIC (Fig. 1A) and those using SP catheters (Fig. 1B). Pre-mapping bladder outcomes (open circles) from urodynamic testing in the CIC group revealed average bladder capacity within normative ranges, per ICS guidelines (300–600 mL, optimal ranges in lower right quadrant)^42^. However, average detrusor pressure and systolic blood pressure values at maximum capacity were elevated above normative ranges (40 cmH_2_O—detrusor pressure, upper quadrants, i.e. 110–120 mmHg—blood pressure, right panels^20,43^). Bladder capacity values for the SP group were below normative storage values for 2/3 participants. Maximum detrusor pressure and systolic blood pressure values were elevated above normative ranges for all 3 participants. BC-scES mapping targeted parameters that promoted an increase in capacity while reducing maximum detrusor pressure and systolic blood pressure responses to bladder distention for both groups. The measurement trends for each participant are illustrated with the ellipses.

**Figure 2:**
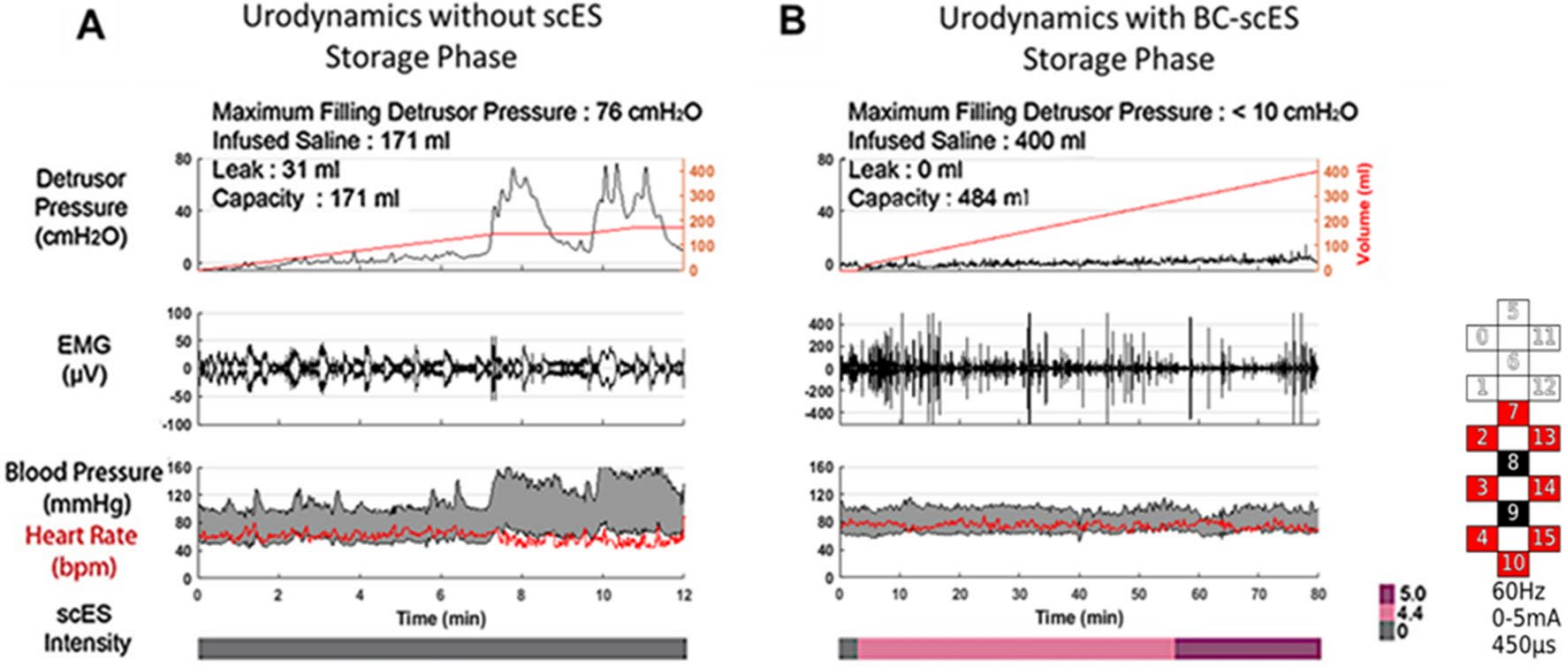
Improvement in bladder compliance using targeted scES parameters (BC-scES). (**A**) Example of detrusor pressure (black, top panel) and bladder capacity (red, top panel), sphincter EMG (µV, middle panel), blood pressure (mmHg, black, systolic—top line, diastolic—bottom line, lower panel) and heartrate (red, lower panel) in the absence of scES in a participant with chronic SCI (B24); Note the detrusor overactivity with incontinence at low capacity, and a simultaneous rise in systolic blood pressure; (**B**) in the same participant using BC-scES and parameters adjusted for bladder compliance. Maintenance of bladder compliance (increased bladder capacity without a change in detrusor pressure in response to bladder filling) was intensity (V, pink bar) dependent and participant- specific. Electrodes: cathodes = black; anodes = red; inactive = white.

A representative example of the detrusor pressure–volume relationship, sphincter electromyography (EMG) responses, and systolic blood pressure without scES and with targeted BC-scES is provided in Fig. 2A,B, respectively. Without scES, detrusor responses to increased bladder volume exhibited instability marked by sharp and sustained increases in detrusor pressure or neurogenic detrusor overactivity (Fig. 2A). Additionally, detrusor pressure rose above clinically-recommended thresholds^42^ for bladder filling (> 10 cmH_2_O) and detrusor leak-point pressures (> 40 cmH_2_O). Furthermore, timed with each non-voiding contraction was an increase in systolic blood pressure, which remained elevated and outside the normative reference range (i.e. 110–120 mmHg^20,43^), resulting in cessation of bladder filling, removal of residual volume, and a subsequent return to pre-fill arterial pressure values. Such instability in both systolic blood pressure and detrusor pressure limit bladder compliance, as evidenced by repeated reflexive contractions resulting in incontinence. Note, participants’ blood pressure and heart rate were closely monitored during testing as well as signs and symptoms of autonomic dysreflexia. There were no complications as a result of higher blood pressures during urodynamics. Following BC-scES mapping, stable detrusor filling pressure (< 10 cmH_2_O) with increased volume representative of improved bladder compliance and increased sphincter EMG activation for maintenance of urinary continence was achieved (Fig. 2B). Systolic blood pressure also remained stable (i.e. 110–120 mmHg) with optimal BC-scES.

**Figure 3:**
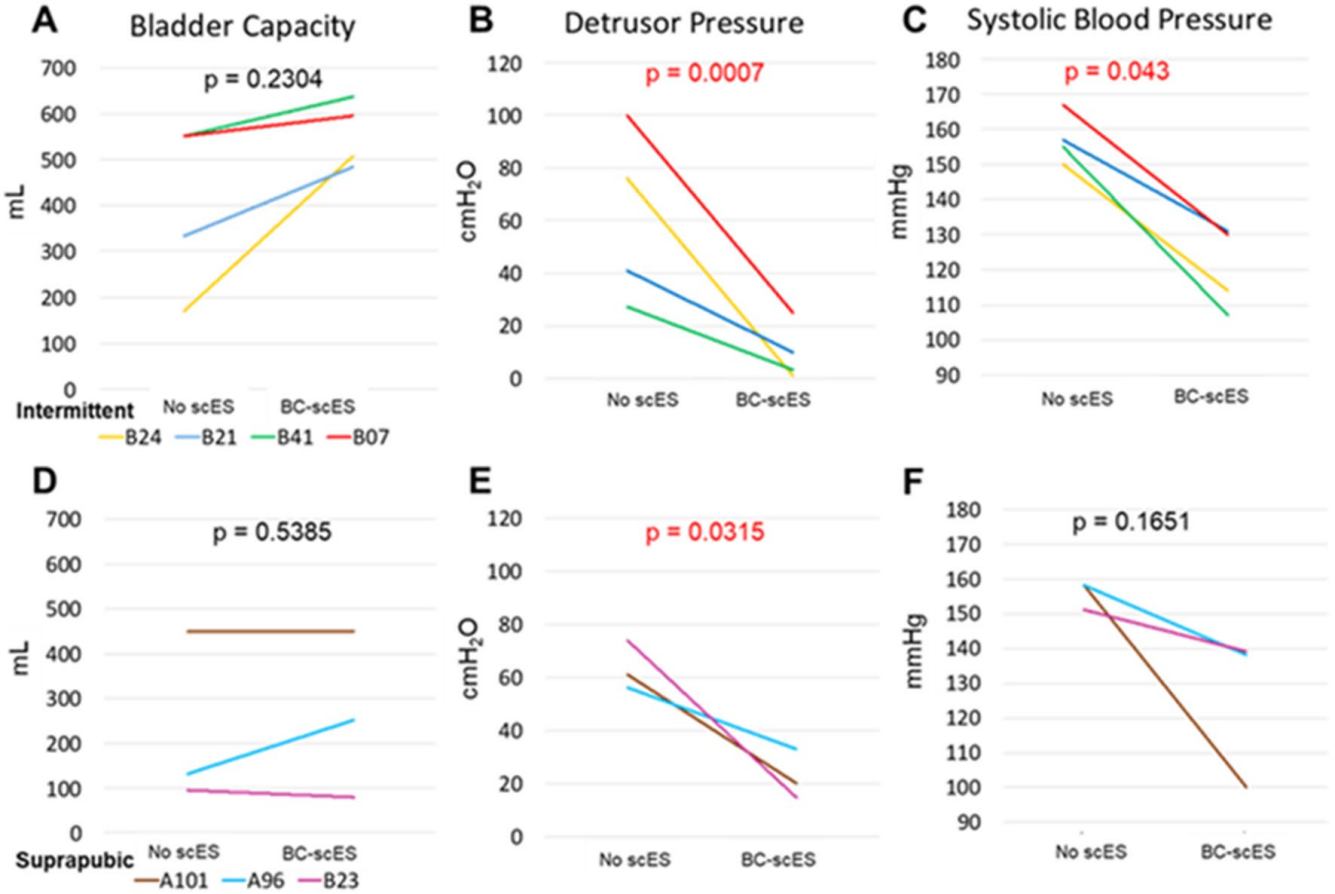
Comparison of bladder capacity, detrusor pressure and systolic blood pressure without scES relative to optimized BC-scES parameters for participants intermittently catheterizing, n = 4 (**A**–**C**); and participants with a suprapubic catheter, n = 3 (**D**–**F**). BC-scES mapping significantly improved (reduced) detrusor pressure and systolic blood pressure at maximum capacity in those using intermittent catheterization and detrusor pressure in the suprapubic group.

Optimal BC-scES parameters that improved bladder capacity, while reducing maximum detrusor pressure and systolic blood pressure responses to bladder distention were compared across participants relative to outcomes obtained without scES (Fig. 3A–F). Post-mapping optimal BC-scES parameters in the CIC group resulted in significant improvements (reduction) in maximum detrusor pressure (p = 0.0007) and maximum systolic blood pressure values (p = 0.043) relative to no scES (Fig. 3B,C). Post-mapping optimal BC-scES parameters in the SP group resulted in a significant reduction in detrusor pressure relative to no scES (p = 0.0315) (Fig. 3E). Refer to Table 2 for the percent change in each outcome for both groups. Note that the effective BC-scES parameters were not always from the final mapping session. In all participants, improvements in detrusor pressure were achieved with high-frequency configurations (i.e. > 60 Hz).

**Table 2:** Comparison of bladder mapping group outcomes without scES and with targeted BC-scES.

Cystometry measurements at maximum capacity	CIC				SP			
	No scES	BC-scES	% Change	p-value	No scES	BC-scES	% Change	p-value
**Bladder capacity (mL)**								
Avg. ± S.D.	401 ± 184	563 ± 79	66.4 ± 88.5	0.2304	226 ± 195	308 ± 221	24.2 ± 57.1	0.5385
Range	171–450	483–637			96–450	120–552		
**Detrusor pressure (cmH**_**2**_**O)**								
Avg. ± S.D.	61 ± 33	14 ± 20	− 84.5 ± 11.4	0.0007*	64 ± 9	28 ± 5	− 62.7 ± 19.7	0.0315*
Range	27–100	1–43			56–74	23–33		
**Systolic blood pressure (mmHg)**								
Avg. ± S.D.	157 ± 7	121 ± 13	− 23.4 ± 5.9	0.0043*	156 ± 4	139 ± 15	− 19.1 ± 15.4	0.1651
Range	150–167	107–133			151–158	125–155		

*Indicates significance. *Avg.* average, *BC* bladder compliance, *CIC* clean intermittent catheterization, *cmH*_*2*_*O* centimeters of water, *mL* milliliters, *mmHg* millimeters of mercury, *scES* spinal cord epidural stimulation, *S.D.* standard deviation, *SP* suprapubic catheter.

### Bladder mapping—emptying phase

**Figure 4:**
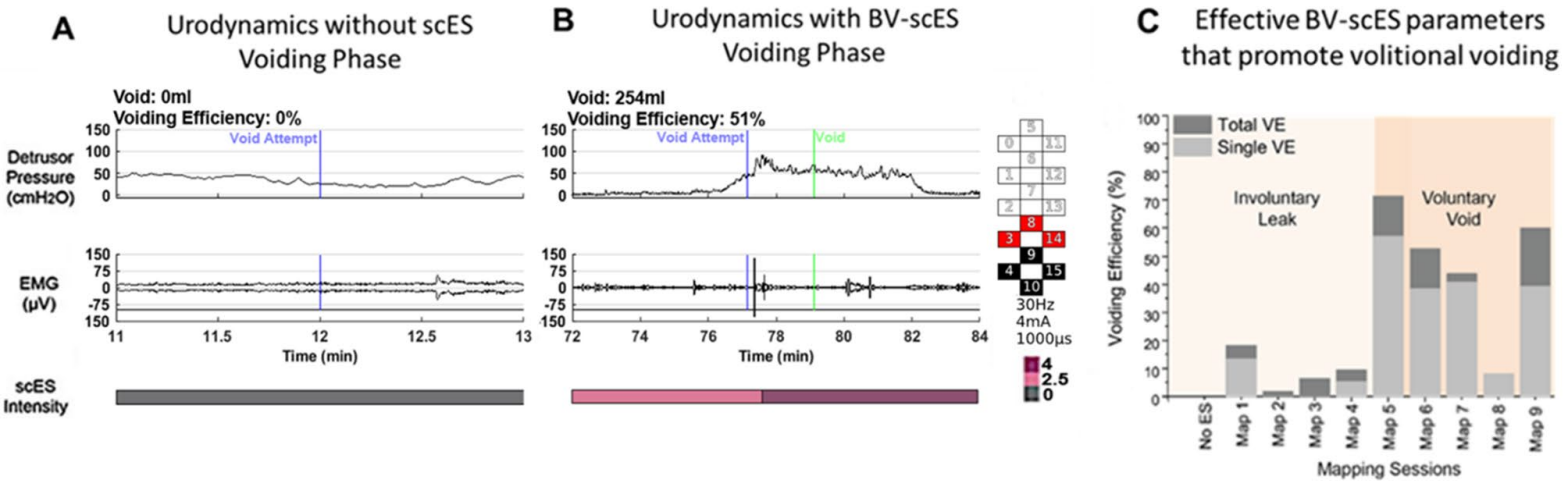
Improvement in ability to initiate bladder voiding with targeted BV-scES. Example of detrusor pressure (upper panel) and sphincter EMG (µv, lower panel) in the absence of scES (**A**) in an individual with chronic SCI (B07) (Leak: 0 mL; Capacity: 622 mL); (**B**) Representative cystometry recording in the same individual using BV-scES and parameters adjusted for void initiation (VE: 51.2%, Capacity: 496 mL); Voiding initiation was intensity (pink bar) dependent and participant-specific. Note the rise in detrusor pressure timed with relaxation of sphincter EMG activity and a return of detrusor pressure to baseline. (**C**) Effective and non-effective BV-scES parameters for promoting volitional voiding during urodynamics mapping sessions for B07. Initiating a void occurred only in the presence of optimized BV-scES. Reflexive leaks are indicated as involuntary. Light gray indicates the voiding efficiency (VE) for a single leak/void and dark gray indicates total voiding efficiency for a mapping session when multiple void attempts were possible. Mapping sessions were approximately 1 week apart. VE = [Void Amount/Void + Residual] × 100; Electrodes in B: cathodes = black; anodes = red; inactive = white.

Subsequent mapping for bladder void initiation (BV-scES) was evaluated during filling cystometry at 80% of leak point volume. Voiding was not achieved without scES in any of the participants. An example cystometry recording of the void attempt without scES is shown in Fig. 4A. The initiation of voiding with scES was achieved in participants when timed to intent and the desire to void with the sensation (direct or indirect) of bladder fullness (example, Fig. 4B), demonstrating the generation of a detrusor contraction and concurrent relaxation of the sphincter during voiding. Note, that the intensity ramp-up was timed with the participant’s sensation and report of the desire to void. Importantly, the void is timed close to the initiation of the attempt, generating a detrusor contraction from a low-pressure baseline and subsequent return to baseline after voiding. Effective BV-scES parameters were sufficient to generate the initiation of voiding with varying degrees of voiding efficiency (Fig. 4C). Voiding efficiency differences were a result of involuntary reflexive bladder contractions (Maps 1–4) relative to when the initiation of voiding was timed to participant sensations of bladder fullness the desire to void (Maps 5–9). BV-scES mapping identified configurations that were frequency-dependent, and distinct from BC-scES, with the initiation of voiding occurring at low frequencies, between 25–30 Hz, for 6/7 participants.

### Targeted regions of the spinal cord with scES during bladder storage and voiding

**Figure 5:**
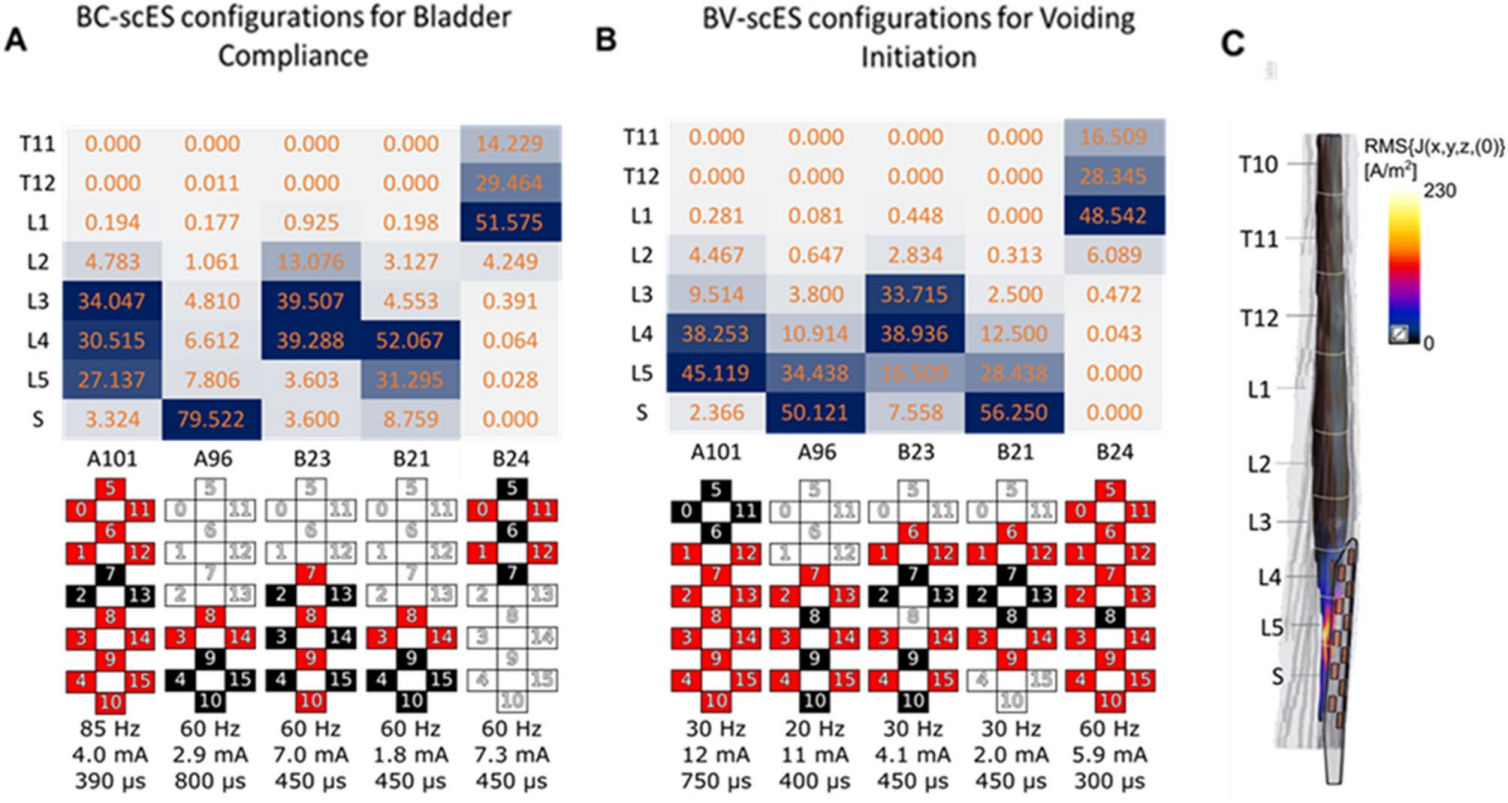
Heatmap plots of the amount of percent total electric charge delivered across each segment that resulted in best bladder storage outcomes (**A**) and bladder voiding (**B**) for each individual (x-axis) and the areas of the spinal cord that were directly targeted by the stimulation (y-axis) as well as the corresponding map configurations (black—cathodes; red—anodes) and stimulation parameters; (**C**) Example of MRI-based 3D model of the spinal cord at lumbosacral enlargement and location of scES paddle electrode with respect to the spinal cord levels. Distribution of electric current density is highlighted with heatmap. Simulations are performed using Sim4Life platform. *BC* bladder compliance, *BV* bladder voiding, *L* lumbar, *S* sacral, *scES* spinal cord epidural stimulation, *T* thoracic.

The maximum amount of electric charge per second delivered to the spinal cord and corresponding levels targeted by effective BC-scES and BV-scES parameters (for participants with available high-resolution MRI data) are illustrated in Fig. 5A,B, respectively. Activation of the rostral (spinal cord level L1) to mid-lumbosacral enlargement (spinal cord levels L3–L4) overlapped for 4/5 participants using effective BC-scES, while activation of the caudal (sacral) region of the lumbosacral enlargement was effective for bladder compliance for 1 participant (Fig. 5A). Activation of the caudal region of lumbosacral enlargement (spinal cord levels L4–S1) using BV-scES overlapped for 4/5 participants (Fig. 5B). Less charge was required for a void effect relative to a storage effect. An example of an MRI-based 3D model of the spinal cord at the lumbosacral enlargement for participant B21 and the location of the scES paddle array with respect to the spinal cord is shown in Fig. 5C, where the distribution of the current density targets the caudal region of the lumbosacral enlargement.

## Discussion

The current study investigated the effects of scES on bladder function through targeted mapping during filling cystometry. Placement of electrodes and the direction/extent of current spread on the spinal cord targeted by scES bladder cohorts was also performed using high-resolution MRI and computational modeling to better understand the neuroanatomical regions responsible for mediating improvements in LUT function. For those individuals with available high-resolution MRI (n = 5), neuromodulation of bladder compliance was primarily effective when scES targeted spinal cord regions L3–L4, while initiation of voiding was enhanced at caudal regions of the lumbosacral spinal cord (L4–S1). Storage and void effects were frequency-dependent, with high-frequency cohorts mediating bladder compliance and low-frequency cohorts mediating void initiation. Intensity selection for storage and voiding was individual-specific.

Epidural stimulation targeting the lumbosacral spinal cord has been shown in multiple studies to improve bladder function in humans with chronic SCI, even when stimulation was not directly optimized for bladder function^21,22,25,26,44^. Our previous work demonstrated that LUT function benefits from combined activity-based recovery training with scES targeted for either stepping/standing and cardiovascular/voluntary movement^21^. While scES was not directly configured for bladder function, nor was stimulation “on” during cystometry, optimizing the state of excitability of the human spinal circuitry with scES and through the integration of appropriate sensory information with task-specific training, may have led to improved adaptations in detrusor activity and reciprocal somatic facilitation of the sphincter. However, blood pressure was not entirely stabilized in response to bladder distention. Here we show that scES targeting bladder compliance also maintained systolic blood pressure within normative ranges during cystometry, likely due to suppression of detrusor overactivity at larger storage volumes.

Assessment of urodynamic parameters during BC-scES mapping revealed improvements in detrusor filling pressure and maintenance of tonic sphincter EMG activity, which resulted in improved bladder compliance and stabilization of systolic blood pressure that was most effective in the upper to mid lumbar regions, with L3–L4 segments appearing to be a key region. The activity of the detrusor muscle and external urethral sphincter (EUS) can be coordinated and modulated by neural circuits located in the spinal cord as well as from supraspinal centers^45^. Pre-clinical research in rodents suggests evidence of a lumbar coordinating center at L3–L4 spinal segments that contributes to the emergence of EUS bursting and detrusor-sphincter coordination after SCI^46,47^. Similarly, neurons involved in mediating the ejaculation reflex, which involves periurethral muscles important for sphincter coordination and anterograde semen propulsion, have been identified in the L3–L4 spinal segments in rats^48^ and from L3–L5 in humans^49^. Furthermore, activation of the dorsal surface of the spinal cord with stimulation at L3 in rodents has been shown to reduce detrusor overactivity^50^ and effectively modulate EUS activity, reducing urethral resistance and promoting emptying^51,52^.

Activation of the rostral array targeting upper lumbar sympathetic (L1–L2) regions of the spinal cord may also help facilitate urinary storage, promoting low intravesical pressures and relaxing the detrusor muscle during bladder filling. As the bladder accommodates a larger volume, afferent fiber activation initiates an intersegmental spinal reflex pathway from the sacral cord to thoracolumbar sympathetic neuron, which stimulates contraction of the internal urethral sphincter and inhibits bladder activity^53^. The storage effect is enhanced, as intrinsic viscoelasticity of the detrusor muscle permits the bladder wall to accommodate increasing volume, while the parasympathetic pathway remains quiescent^54^.

In one participant (A96), a positive storage effect was achieved by stimulation at the conus. Somato-visceral sacral reflexes that remain intact after suprasacral SCI can be effectively targeted to neuromodulate bladder function. Stimulation-induced activation of pudendal afferents, which project onto sympathetic and parasympathetic pathways can lead to inhibition of the detrusor muscle, suppression of overactivity, and simultaneous excitation of the sphincters, resulting in a coordinated storage response^55^. Indeed, numerous studies in humans with SCI have shown that stimulation of branches of the pudendal nerve suppresses bladder hyperreflexia, reducing incidences of incontinence and improving bladder capacity^56–61^. Similar to mechanisms involved in pain control with spinal cord stimulation^62,63^, antidromic facilitation of somatic fibers to promote a sphincter guarding response, as well as orthodromic activation of ascending sensory dorsal column fibers to upper and mid-lumbar segments, including interneuronal segmental connections, may contribute to the storage effect with scES at distal segments.

The results of the present study also expand the scope of our earlier investigations in which we identified scES parameters that improved the efficiency of the reflexive void^22^ after motor complete SCI. Similarly, we identified low-frequency BV-scES parameters that were used to augment the intent to void and were driven by sensations of bladder fullness at 80% of leak point volume. Each participant was able to initiate voiding in the presence of scES. Lumbosacral BV-scES may be used to enable the spinal cord, below the level of injury, to efficiently integrate afferent sensory information from the bladder, along with residual signals from the pontine micturition center, to generate inhibitory input to sympathetic and somatic regions of the spinal cord^64^. The initiation of voiding was most effective at caudal segments of the spinal cord where local spinal reflexes involved in sphincter coordination could be modulated to decompress the sphincter during intent and with sufficient bladder volume, simultaneously facilitate parasympathetic activation of the detrusor muscle^65^. During void attempts, an increase in intra-abdominal pressure (indirectly measured via the rectal catheter during urodynamics) may assist with decompression of urethral resistance at the bladder neck and proximal urethra to initiate emptying. It’s important to note that the initiation of voiding began from low detrusor pressure and while detrusor pressure rose during void attempts and was sufficient to override the pressure generated from urethral sphincters, it did not remain sustained, which could result in vesicoureteral reflux. While the focus of this study was to initiate voiding, future work is aimed at implementing BV-scES training to improve the efficiency of the void. Even though inhibition of EUS activity during micturition is partly dependent on supraspinal mechanisms, once the initiation of urine flows through the urethra, voiding is facilitated by a urethral-to-bladder reflex and increased efferent excitatory outflow to the bladder via pelvic nerves^66^. BV-scES training can be used to strengthen these local circuits.

Furthermore, as demonstrated in our findings, scES can benefit multiple systems synergistically. Darrow et al. demonstrated the application of motor-scES subsequently resulted in participant-reported gains in bladder and bowel management, sexual function, and cardiovascular responses to orthostatic challenges, although scES mapping targeting the different autonomic systems was not performed^26^. Similarly, Walter et al. demonstrated acute neuromodulation of detrusor pressure and sphincter EMG activity with a reduction in bowel emptying time reported by the participant^25^. In corroboration with our findings, sufficient generation of detrusor pressure was also generated between 30–40 Hz^25^. It should be noted that systematically mapping for the target system is important for understanding how the current physiology can be neuromodulated, consideration of any off-target effects, and whether adjustment of parameters is necessary over time. For example, one case report indicated that scES, which was optimized for motor function, did not benefit bladder function in one individual^67^.

Cardiovascular function is another synergistically impacted system. Cardiovascular dysfunction after SCI, such as autonomic dysreflexia that has been reported to occur up to 40 times a day^68^ in susceptible individuals (primarily SCI above T6), with neurogenic detrusor overactivity being one of the primary triggers^69^. We, and others, have shown that cardiovascular instability in response to bladder filling is widespread after SCI, also occurring in those with injuries below T6, as the sympathetic outflow extends to L2^21,70,71^. The ability to recover bladder function such as, increasing bladder capacity, minimizing detrusor instability, and improving bladder pressure and emptying, is limited by such severe fluctuations in blood pressure. Regulating blood pressure in those having SCI is challenging and is exacerbated by current methods of monitoring bladder function, which is limited to testing only in a clinical urodynamics laboratory. The extreme dysregulation of cardiovascular and bladder function underscores the importance of addressing these multi-faceted autonomic complications.

Understanding the parameters of stimulation and programming strategies represents an important and necessary step in advancing neuromodulation targeted at improving autonomic function after SCI. Neuroimaging and image-based computational modeling of the spinal cord, nerve roots, and surrounding tissue can optimize outcomes for stimulation-based interventions, such as scES. Our recent study has shown that maximizing the coverage of the excitable tissues by electrical stimulation at relevant levels of the spinal cord can improve functional outcomes^72^. Also, the length of the spinal cord and location of spinal cord segments with respect to the vertebrae, particularly at lumbosacral enlargement, varies across individuals with the location of the conus tip ranging between T12 to L2. Therefore, relying only on the vertebral levels as a guide for stimulating specific spinal cord regions could often be inadequate and not lead to the best possible outcomes, limiting the understanding of the mechanism of action of electrical stimulation as a neuromodulation intervention. The differences in spinal cord level of activation across participants may also be due to the level at which the electrode array was placed and available tissue accessible for scES. It is also unknown if training with parameters specifically configured to modulate urinary continence and emptying is necessary to achieve maximum benefit for bladder function.

Given that scES involves a more invasive surgical implantation procedure, limiting its scope, other, less invasive approaches that utilize cutaneous or percutaneous placed electrodes targeting either spinal cord segments or peripheral nerves, may extend neuromodulatory approaches for bladder function in SCI. Selective electrical stimulation of the genital nerves (branches of the pudendal nerve), which can be used in the clinic or home setting, has been shown to improve urinary continence in multiple clinical studies^56,59,60,73–78^. Similar to the findings of the current scES study, stimulation amplitudes can be adjusted to participant reported sensations (e.g. urgency) to further maintain continence^76^. Other similarities related to frequency selection across clinical and pre-clinical studies indicate an optimal range for activation of the micturition reflex to promote a void response (increase in detrusor pressure and simultaneous sphincter EMG relaxation) between 20–40 Hz, which is dependent on the state of the bladder with sufficient intravesical volume of fluid^22,79–83^. A potential mechanism for a frequency-dependent response is similar to that proposed for differential motor output behaviors (rhythmical, step-like movements of the lower limbs^16,84^ or sustained lower limb extension^85^), supporting the concept that coexisting pathways can be differentially modulated according to the “central state” of the spinal cord^86^. It is important to note that potential differences in optimal frequency selection between studies can be due to tissue differences related to spinal network activation versus selective stimulation of nerve fibers and the relationship of action potential conduction velocities (central vs peripheral nervous system). While frequency ranges may differ from peripherally- to centrally-based stimulation approaches, the primary effects of stimulation likely occur through the modulation of the intact spinal circuitry below the level of injury and the relative physiological state of the bladder with the potential for descending input from supraspinal centers.

While the anatomical variability across participants is important to consider during scES mapping approaches, the amount of testing and optimization performed in the laboratory is likely unfeasible in a clinical setting. Decision-making processes of scES for bladder should consider monitoring real-time responses from both the internal (pressure, cmH_2_O) and external urethral sphincters (EMG, µV—note, for feasibility, the external anal sphincter is used as a clinical correlate) to isolate initial parameters for continence and void initiation. Registration of an immediate response from vesical pressure to scES is challenging given the viscoelastic properties of the detrusor and accommodation of fill volume. Furthermore, electrode steering can be used to drive scES to enhance either a storage effect or promote a void effect, as precise placement of the paddle array relative to the sacral micturition center can be difficult due to inherent limitations of electrode design in obtaining both full lumbar and sacral cord coverage.

## Conclusion

The results of the current study demonstrate that scES can be used to simultaneously and safely modulate urinary continence and the initiation of voiding while managing distention-associated dysregulation of blood pressure. Importantly, these initial findings reveal the complex dynamics and interplay between sympathetic and parasympathetic circuitries that are being integrated and regulated within the spinal cord below the level of SCI. This spinal circuitry is driven by afferent input and modulated by scES to effectively optimize the state of the bladder and associated systemic blood pressure responses. It is also likely, given the void intent results, that scES enhances the conduction properties of residual damaged or non-functional but anatomically intact long ascending/descending axons that are traversing across the spinal injured segment. In this manner, scES acting upon lumbosacral spinal neural networks can promote an increase in overall autonomic regulation sufficient to interact with appropriate sensory cues (e.g. from bladder distention) as well as engage descending supraspinal residual inputs (e.g. intent to void) to facilitate continued involvement of such networks to maintain target bladder compliance, initiate on-demand voiding, and regulate cardiovascular parameters during storage and emptying. Current studies in progress are evaluating the integration of BC-scES and BV-scES in the home setting in order to understand the natural transition from storage to voiding.

## Limitations

As mentioned in the “Results” section, two participants did not receive high-resolution MR imaging using our established imaging protocol that highlights the lumbosacral enlargement, which precluded the ability to generate a 3D model of the spinal cord and subsequent scES simulations. Anatomical variability in the size (length, area, volume) of the lumbosacral spinal cord across individuals may also result in differing mechanisms of action of scES. Similar findings have previously been reported in studies related to the use of spinal cord stimulation for pain^87,88^. The same electrode combinations may enable different neurophysiological outcomes across individuals due to activation of different spinal cord regions and spinal cord networks. Importantly, the extent, severity, mechanism of the injury, number of residual fibers as well as clinical and demographic factors may also influence the neuromodulatory effects of spinal cord stimulation.

## Methods

### Participants

Seven individuals (32.1 ± 4.6 years of age; 6:1, male:female) with motor complete SCI (C3-T2) participated in a research study conducted at the University of Louisville investigating the effects of scES directly targeted to improve bladder storage and emptying (Institutional Review Board #17.1024, NCT03452007, Task and Physiological Specific Stimulation for Recovery of Function after Severe Spinal Cord Injury: Functional Mapping with Lumbosacral Epidural Stimulation for Restoration of Bladder Function after Spinal Cord Injury) between the years of 2018–2021. As part of another study conducted at Frazier Rehabilitation Institute, participants were already surgically implanted with a 16-electrode array (5–6–5 Specify, Medtronic, Minneapolis, MN, USA) at the T11–L1 vertebral levels over spinal cord segments L1–S1 as previously described^13,15^. The electrode lead was tunneled subcutaneously and connected to the pulse generator (RestoreADVANCED (B21, B23), or Intellis (A101, A96, A68, B24, B07), Medtronic, Minneapolis, MN) placed ventrally in the abdomen. All research participants were over 21 years of age at the time of scES implant and met the following inclusion criteria: non-progressive SCI at the cervical and upper thoracic spinal cord, AIS A or B, and at least 2 years post-injury with no medical conditions unrelated to SCI at the time of implant. The initial time frame of enrollment in this study following implant surgery was 3.3 ± 2.8 years. All research participants provided written, informed consent and the research was approved by the University of Louisville Institutional Review Board. All research was performed in accordance with relevant guidelines and regulations.

### Clinical evaluation

Participants received a clinical evaluation prior to study participation to assess motor and sensory status. Two clinicians independently performed the International Standards for Neurological Classification of Spinal Cord Injury^89,90^ in order to classify participants’ injuries using the ASIA (American Spinal Injury Association) Impairment Scale (AIS). A physical examination and bladder/kidney ultrasound were performed by the study physician and study urologist, respectively, for medical clearance, ensuring participation safety using the following inclusion criteria: (1) stable medical condition; (2) no painful musculoskeletal dysfunction, unhealed fracture, contracture, pressure sore or urinary tract infection that might interfere with training; (3) no untreated psychiatric disorders or ongoing drug abuse; (4) clear indications that the period of spinal shock is concluded determined by the presence of muscle tone, deep tendon reflexes or muscle spasms and discharged from standard inpatient rehabilitation; (5) non-progressive supra-sacral SCI; (6) bladder dysfunction as a result of SCI; and (7) epidural stimulator implanted at the lumbosacral spinal cord. None of the participants had ever received Botox injections for management of bladder dysfunction and all participants were off anti-spasticity medication (e.g. Baclofen). None of the participants altered their method of bladder emptying throughout the study.

### Urodynamics

All data were obtained from standard urodynamic evaluations with recommendations from the International Continence Society^42^. Using the Aquarius^®^ LT system (Laborie, Williston, VT), cystometry was performed in the seated position via a single sensor, dual-channel catheter (7 Fr, T-DOC^®^ Air-Charged™, Laborie, Williston, VT) with the continuous filling of sterile, body-temperature saline (37 °C) at a fixed rate of 10 mL/min, more closely reflecting physiological filling. Abdominal pressure was measured via a rectal catheter (7 Fr, T-DOC^®^ Air-Charged™, Laborie, Williston, VT). Pelvic floor EMG (Neotrode II, Laborie, Williston, VT) was recorded using surface patch EMG electrodes and a grounding pad was placed on a bony prominence, usually the hip or knee. Note that to distinguish between isolated EMG activation of the intramuscular urethral striated sphincter versus general muscle activation of the pelvic floor, an intramural needle electrode EMG is necessitated. However, given the ethical concerns of repeated needle electrode placement, surface electrodes are consistently used in daily clinical practice as an established method for diagnosis of lower urinary tract dysfunction^91^. Detrusor pressures were calculated by subtracting the intra-abdominal pressure from the intra-vesical pressure. Research participants were asked to cough to verify catheter positions. Prior to the start of filling, scES amplitude was ramped up slightly to isolate the initial targeted location (pelvic floor, bladder, abdominal region versus legs, feet—see “Bladder Mapping” section for details below). During the filling phase of the experiment, participants were instructed to communicate bladder sensations (first sensation); the desire to urinate (first urge to void); and the strong desire to void, and the feeling that voiding/leaking cannot be delayed (maximum capacity). Given that many SCI participants may have a loss of bladder sensation, indirect sensations were also used. The volume of water infused and bladder pressure was continuously recorded. Uninhibited bladder contractions also were identified. Blood pressure (BP) and heart rate (HR) were obtained from the brachial artery, and measured by oscillometric technique (Carescape V100, GE Healthcare, Milwaukee, WI), throughout the urodynamic session. Baseline BP recordings were obtained in the supine and seated positions prior to urodynamic testing. Any signs and self-reported symptoms of autonomic dysreflexia were documented and observed throughout testing. Bladder filling was ceased and the bladder was emptied if any of the following conditions were observed: (1) spontaneous urine leakage, (2) filling ≥ 600 mL or reaching maximum bladder capacity as evidenced by a rise in the compliance curve, (3) high sustained intravesical pressure ≥ 40 cmH_2_O or, (4) autonomic dysreflexia as evidenced by a sustained systolic blood pressure recording of ≥ 20 mmHg from baseline and/or intolerable symptoms. A post-fill BP recording was captured to ensure BP values returned to baseline.

During the voiding phase, a “permission to void” command followed after stopping the infusion pump (at approximately 80% of leak point volume). Detrusor pressure was monitored during the void attempt and uroflowmetry for the voided volume. Post-void residual volume was measured to evaluate the extent of bladder emptying. Importantly, natural diuresis occurs during cystometry and may contribute to measured bladder volumes^92^. Note, that voiding was not attempted if blood pressure and detrusor pressure were elevated, as indicated above.

Bladder capacity was calculated as the volume of leaked or voided fluid plus any residual amount removed from the bladder. Voiding efficiency (VE) was calculated as: VE = [volume voided/(volume voided + residual volume) × 100]. Compliance was calculated by dividing the volume change (ΔV) by the change in detrusor pressure (ΔPdet) during that change in bladder volume and was expressed in mL/cmH_2_O. The intravesical pressure (Pves) at which involuntary expulsion of water/urine from the urethral meatus was observed was considered the detrusor leak point pressure (DLPP). Maximum detrusor pressure (MDP) was identified as the peak detrusor pressure during the voiding phase of the cystometrogram. Detrusor pressures were calculated by subtracting the intra-abdominal pressure from the intra-vesical pressure. Note, if a participant did not leak during the filling cycle, MDP was used in place of DLLP. All analyses were performed with customized software in MATLAB (MathWorks, Natick, MA, 2017A).

### Bladder mapping

Following enrollment, each participant completed a baseline Urodynamics without stimulation, followed by approximately 8 weeks of bladder mapping. Spinal cord epidural stimulation (scES) was administered by a multi-electrode array implanted in the epidural space over the dorsum of the spinal cord. An implanted package containing stimulating circuits, a rechargeable battery, and wireless communication activates the electrodes (16 platinum electrodes arranged in three columns of [5–6–5], Medtronic Inc.). The pattern of electrically active electrodes, as well as electrode voltage, stimulating frequency, and stimulating pulse width was remotely programmed. Since different spatial activation patterns and different frequency parameters affect different spinal circuits, the electrode array was reconfigured, within limits, to bias its facilitating effects toward bladder storage and emptying. Bladder mapping followed a human-guided interactive optimization approach^93^ where the experimental mapping process was subdivided into separate domains/tasks to isolate parameters for storage function and the initiation of voiding. Since these domains are inter-dependent, subsequent optimization tested and refined parameters concurrently in order to build comprehensive cohorts for multi-system stimulation. Each participant completed a minimum of 20 urodynamic sessions (10 for storage; 10 for void initiation) mapping the detrusor and urethral pressure responses as well as sphincter EMG responses during both filling and emptying cystometry phases while scES parameters (anode, cathode selection; frequency and amplitude, and the number of cohorts) were modulated to isolate successful configurations. The goal for bladder capacity (BC)-scES was to target volumes between 400–500 mL based on average normal capacity and avoiding over-distention in individuals performing intermittent catheterization 4–6 times/day (including average fluid intake)^42,94^. Also targeted were filling pressures (< 10 cmH_2_O^95,96^) to improve overall bladder compliance and detrusor leak-point pressures (< 40 cmH_2_O)^42^. Maintaining normative systolic pressures during filling, within a range of 110–120 mmHg, was a further goal^20^. All enrolled participants completed prior scES mapping studies for cardiovascular function and thus, the cardiovascular cohort was integrated if blood pressure was elevated. All simulations accounted for any cardiovascular cohorts.

Based on previously published methods^13,22^, bladder mapping was performed by selecting electrode configurations with cathodes positioned caudally, targeting the sacral micturition center and parasympathetic pathways, then with cathodes positioned in the mid-array to target the purported lumbar spinal coordinating center with presynaptic connections to sphincter motoneurons^46,47^, and then with cathodes positioned rostrally, to target sympathetic pathways (location selection order varied for each mapping session). Changes in detrusor pressure, sphincter activation/relaxation, and blood pressure responses were monitored during bladder filling while conducting a gradual ramp-up of stimulation frequency and intensity until a near-motor threshold stimulation amplitude that did not elicit direct lower limb movements was selected. The increase in stimulus amplitude was applied once frequency was fixed for the specific trial, which was at approximately 80% bladder capacity/80% of leak point volume and in response to participant sensations of bladder fullness or the desire to void. The aim was to increase sensory feedback and the participant’s intent during mapping to augment either storage (increase in sphincter EMG and urethral pressure and reduction of detrusor pressure) or void effects (increase in detrusor pressure, decrease in urethral pressure, and quiescence of sphincter EMG activity). The effects of varying frequency at a fixed pulse width (µs) were applied in both an ascending (low to high frequency, 15–90 Hz) and descending order (high to low frequency, 90–15 Hz) in increments of 5 Hz for both BC-scES and BV-scES mapping at the initiation of filling cystometry (to register changes in sphincter EMG activity) and at 80% capacity/80% of leak point volume (to increase the storage or void effect). Void initiation was also attempted without stimulation. Note that, lower frequencies (e.g. 5 and 10 Hz) oftentimes elicited excessive lower extremity activity. This procedure was applied across all participants and trials. Stimulation frequency and intensity were then modulated synergistically in order to isolate an optimal frequency that elicited an overall continuous low detrusor pressure filling profile with a synchronized sphincter EMG pattern effective for bladder continence. Guided by participants’ sensations of bladder fullness, the transition from continence to micturition aimed to integrate ascending inputs and descending volitional drive. Electrodes in the caudal, middle, and rostral regions of the array were selected while the frequency was kept fixed and amplitude adjusted in order to isolate an optimal intensity that drove the initiation of voiding activity (simultaneous increase in detrusor pressure with a decrease in upper urethral pressure and quiescence of sphincter EMG responses). Electrode location and selection refinement was further modified to adjust for sensory and autonomic symptoms during mapping.

To determine the most optimal stimulation configuration among those tested within participants, we quantified the degree of improvement, Imp_coeff_, as a function of bladder capacity, BC, and detrusor pressure, DP, as follows,

$$Imp_{coeff} = \frac{BC}{{BC_{BL} }} \times \frac{1}{{\left( {\frac{DP}{{DP_{BL} }}} \right).}}$$

An increase in bladder capacity and a decrease in detrusor pressure compared to pre-intervention values will lead to an increase in the improvement coefficient, with a value greater than 1 being improved function, less than 1 being a decrease in function, and 1 suggesting no overall change in function. The results shown in Figs. 3 and 5 were obtained from configurations with the highest improvement coefficient within each participant.

Ellipses were calculated using quartiles determined by a chi-square distribution with a 0.95 confidence interval after outliers more than 1.5 interquartile ranges below the lower quartile and above the upper quartile were removed for both bladder capacity and detrusor pressure.

Lower extremity and trunk EMG was monitored continuously throughout mapping to identify those parameters that modulate detrusor pressure and coordination with the external anal sphincter muscle (mirroring external urethral sphincter) and blood pressure but do not elicit motor activity in the lower extremity or trunk. Stimulation amplitude was lowered and electrode selection was modified to inhibit lower extremity/trunk activity. Note that lower extremity EMG is being analyzed as part of another manuscript addressing off-target effects of scES and thus, analysis was not included in this paper. EMG was collected at 2000 Hz using a 24-channel hard-wired AD board and custom-written acquisition software (Labview, National Instruments, Austin, TX, USA). EMG (MotionLab Systems, Baton Rouge, LA, USA) from the soleus, medial gastrocnemius, tibialis anterior, medial hamstrings, rectus femoris, and vastus lateralis using bipolar surface electrodes with fixed inter-electrode distance. In addition, two surface electrodes were placed over the paraspinal muscles, symmetrically lateral to the epidural electrode array incision site. These two electrodes were used to record the stimulation artifact from the implanted electrode. All mapping urodynamic sessions were conducted at least two days apart.

### High-resolution spinal cord MRI

MRI 2-D scans of all levels of the spine with high spatial resolution were recorded using either Siemens 3.0 Tesla Magnetom Skyra or Siemens 1.5 Tesla ESPREE in sagittal and axial planes. Sagittal images were obtained in two or three separate sequences (depending on the height of the participant) to cover the whole spine from the foramen magnum to the end of the sacral region. These images were reviewed by the radiologist and neurosurgeon to screen for syrinxes, significant stenosis, scoliosis, level of injury and stabilizing treatment, and related surgical changes over time.

Axial images were obtained using T2 Turbo Spin Echo in 4 to 5 separate sequences (depending on the height of the participant) with a focused field of view typically from cervical, upper thoracic, mid thoracic, lower thoracic-upper lumbar, and lower lumbosacral levels. Axial images were obtained with 3 mm slice thickness and zero mm gap. The axial images were used to measure the cross-sectional area of the spinal cord at different vertebral levels and to reconstruct a 3-D individual-specific model of the lumbosacral enlargement necessary for anatomical mapping of the L1–S1 spinal cord segments (described below).

The anterior–posterior and lateral X-ray images of the spinal cord at the location of the scES paddle electrode implant, obtained after implantation from each participant, were used to identify the T12 vertebra based on the location of the last floating rib, and identify the exact location of the rostral and caudal ends of the paddle electrode with respect to the vertebral body. The location of the paddle was estimated with respect to the spinal cord by integrating the lateral X-ray with the sagittal and axial MRI scans^97^. Based on the length of the paddle electrode (46.5 mm for Medtronic Specify^®^ 5–6–5 lead), 15 MRI axial slices (total of 15 × 3 mm = 45 mm in length) that best describe this location were identified. The paddle electrode was placed on the 3-D model based on the location of the identified 15 axial slices.

### Neuroanatomical mapping of lumbosacral spinal cord and computational simulation

The 3-D reconstructed model was completed for 5 out of the 7 participants who had high-resolution MRI scans with incorporation of the finite element modeling technique and neuronal activation function to investigate the distribution patterns of the electric fields generated by scES. Recording MRI axial scans with high spatial resolution enables one to locate and trace the dorsal and ventral nerve roots in the cerebrospinal fluid. The nerve roots that enter the spinal cord at the lumbosacral enlargement elongate to exit the spinal canal further distally at the corresponding vertebral levels (L1–S1). The spinal cord lumbar segments assigned to L1–S1 were anatomically estimated by identifying the set of nerve roots that exit the spinal canal at each vertebral level and back-tracing those nerve roots into the spinal cord body. Furthermore, the axial images of the lumbosacral spinal cord were segmented based on the area of the cerebrospinal canal, spinal cord tissue, and nerve roots. A 3-D model of the spinal cord of each individual was then reconstructed using custom-written codes in MATLAB. The estimated neuroanatomical levels of the spinal cord were visualized on the 3-D reconstructed model of the lumbosacral region.

A set of computational tools were used to map the human spinal cord for bladder function. This toolset included modules from Sim4Life, MANGO, and custom-written programs in MATLAB and Python. Finite element analysis included model creation and generation of the topology and geometry information representing the spinal cord and surrounding tissue boundaries using Sim4Life. The meshing phase decomposed the model geometry into simple shapes or voxels that fill the volume. Each voxel has its own electric field conductivity parameters and initial conditions. Partial differential equations specified electric field distribution among voxels based on the material properties. Features and trends generated from the solutions output from simulations were summarized for each participant. Post-processing produced data products from the instantaneous electric field solution of each stimulation pulse, including visualizations of the fields and current density superimposed on the geometry and along a line traversing the dorsomedial surface of the spinal cord. These results were used to calculate quantiles such as maximums, minimums, averages, and integrals over points. The amount of electric charge per second (in Coulomb per second per square meter) delivered to each level of the lumbosacral spinal cord was calculated by multiplying the amount of current density (ampere per square meter), as determined by the finite element modeling, with the stimulation frequency (hertz) and pulse width (seconds). Data and graphics were exported for illustration and used by MATLAB- and Python-based programs.

### Statistical analysis

Bladder outcomes, all normally distributed per Kolmogorov–Simonov test, were evaluated using paired *t* test. All tests were 2-sided with a significance set to 5%. Statistical analyses were performed in SAS 9.4 (SAS Inc., Cary, NC).

## Data Availability

The datasets generated for this study are available upon request to the corresponding author.
